# Co-producing research study recruitment strategies with and for children and young people for paediatric chronic pain studies

**DOI:** 10.3389/fpain.2024.1358509

**Published:** 2024-07-23

**Authors:** Daniela Ghio, Laura E. Lunt, Angharad Bridges, Lydia Gahr, Anna M. Hood

**Affiliations:** ^1^ Division of Psychology and Mental Health, Faculty of Biology, Medicine and Health, School of Health Science, The University of Manchester, Manchester, United Kingdom; ^2^ Centre for Musculoskeletal Research, Versus Arthritis Centre for Epidemiology, The University of Manchester, Manchester, United Kingdom; ^3^ Manchester Academic Health Science Centre, National Institute of Health Research Manchester Biomedical Research Centre, Manchester University NHS Foundation Trust, Manchester, United Kingdom; ^4^ A National Advisory Group of the Barbara Ansell National Network for Adolescent Rheumatology, Your Rheum, Manchester, United Kingdom

**Keywords:** paediatric pain research, inclusive-recruitment, equality, diversity inclusion, patient and public involvement (PPI), co-development

## Abstract

**Introduction:**

Children and young people experiencing chronic pain are at greater risk of inequitable and poor-quality pain management, which has implications for future management of pain in adulthood. Most chronic pain research is conducted with adults who are more likely to be middle-class, white and monocultured. Inclusive and diverse recruitment practices in paediatric pain research can be an area in which we can address this imbalance of representation. The aim of this current work was to explore these practices and to co-produce recommendations regarding recruitment strategies for paediatric pain research.

**Methods:**

The research team worked with Your Rheum, a United Kingdom young person's advisory group (ages 11–24 years) and diagnosed with rheumatic condition(s), the opportunity to input into rheumatology research. At a virtual Your Rheum meeting, eight young people (female = 7, male = 1, age range 12–24) took part in group discussions, sharing their experiences of taking part in research and their decision process. Online tools, including Mentimeter and Miro, were used to aid conversations and share ideas.

**Results:**

Most young people had experience of taking part in research as a study participant (*n* = 5). Recommendations synthesised included increased awareness of research in general. The young people discussed being open to hearing about research opportunities; they reflected that they are rarely exposed to these invitations or hear about current research. The clinic environment was highlighted as a “good and trustworthy” recruitment area – being approached by a member of the research team was considered ideal, even if it was someone they had not met previously. Many young people recalled little discussions of research at their clinical appointments. Deciding to participate in research included the following considerations: benefit/impact; connecting with others; research topic; which is then balanced against convenience, and reimbursement. The young people felt that taking part in research was empowering and helped them take ownership of their pain management.

**Conclusion:**

It is essential to understand the perspectives of potential study participants, to plan successful recruitment strategies. Ensuring we consider these factors when designing our studies and recruitment strategies is beneficial to all involved. Co-produced recruitment strategies would aid inclusive (and increased) research participation.

## 1 Introduction

Chronic musculoskeletal pain is the third most common cause of chronic pain in children and young people (CYP), with one in five CYP experiencing chronic pain with an incidence rate of 20% (1). The International Association for the Study of Pain (IASP) identified infants and children as one of the key populations at greatest risk of inequitable and poor-quality pain management (2). Poor-quality pain management during childhood contributes to the avoidance of medical care in adulthood (3). As highlighted by the Lancet Child and Adolescent Health Commission, more is needed to make paediatric pain matter, understood, visible, and better to address the paucity of understanding and good quality management of paediatric pain (4). However, most chronic pain research has been conducted with adults who are middle class and white, making it harder to know more about the pain experiences of different cultural, racialized, and socioeconomic groups (4). Children's views are often not represented in research (5). This lack of representation is especially true of young children and CYP from marginalized groups (6) and racialized communities in paediatric pain (4). Therefore, healthcare policies and service development that would be informed by research that is monocultured may mean perpetuating inequities found in paediatric pain.

Ensuring inclusive and diverse recruitment to paediatric pain research can be an area in which we can address this imbalance of representation. There is a misconception that involving younger children from marginalized groups poses both access and communication barriers (7) rather than acknowledging that the systems we use for recruitment are often the barriers to ensuring inclusive involvement and recruitment. Marginalized groups are often pejoratively labelled by researchers as “hard-to-reach” or “non-engaged” without introspection or reflection on why people in these groups may feel unheard and ignored. Justified mistrust due to a historical-exclusion is often not considered (8). The responsibility for ensuring equitable access and engagement with research is with the research team, not the participants. Additionally, a lack of diversity in research teams with associated racialized and gender biases can shape every level of research, from the development of the research question to the diversity of the sample. Inclusive involvement in research has significant mutual benefits for CYP, researchers, and research systems (e.g., funding bodies) by providing evidence that will inform healthcare policies and service development (9, 10).

As Preston et al. (11) highlighted, many of the significant challenges to conducting paediatric health research can be met with solutions from CYP involvement in research development and design. Critically, early CYP involvement with clear communication and goals and meaningful benefit for the CYP is essential to research that can achieve desired outcomes and have long-term impact. There is a growing recognition that children and young people (CYP) should be included in their healthcare decision-making and research development (12). Patient and public involvement (PPI) is an umbrella term given to describe such inclusion in the United Kingdom, whereby involvement occurs across a spectrum with potentially different levels of involvement, from inform, consult, involve, collaborate and empower (13). The United Nations Convention on the Rights of the Child (14), indicates that children (defined as those under the age of 18), have a right to have their voices heard and listened to and a right to express their opinion. This right extends across all aspects of society and, in particular, healthcare access and treatment. However, involving children in their health decision-making and through PPI has only recently been adopted by more research teams (15).

For CYP involvement to be successful, merely having a seat at the table is not enough and it needs to be well-structured and communicated in simple and clear terms (16). The Lundy Model highlights that children must be given inclusive opportunities (e.g., according to their time and priorities), the time and space to express their views and that these views must be listened to and acted upon appropriately (16). The involvement of CYP in research can and should be at all stages of the research cycle, (i.e., priority setting to dissemination) built on a model that falls along the continuum of consultation (e.g., asking for their views), collaboration (e.g., active partnership in research design and process) or user-led (e.g., CYP are the decision makers and not researchers) (17). The benefits of CYP involvement are clear with increased recruitment and retention, which is likely due to the potential challenges and barriers to participation clearly discussed and incorporated into the research design through collaboration with CYP with lived experiences (11). For the Canadian National Standards on Paediatric Pain Management, the working group included patient and family members to contribute to the development providing the necessary point of view of lived experiences (18). There is evidence to showcase that working together with CYP is welcomed by children and researchers (19). Specific replicable guidance is needed to make recruitment for paediatric pain research accessible and inclusive.

Little is known about the decision-making process in taking part in CYP in pain research and if recruitment strategies currently used map onto what CYP want from research. When CYP are included in research, it is often in the development of a specific, already identified research question. When done well, this involvement is collaborative and empowering and can help to shape outcomes in ways that are meaningful for people with lived experience. Our goal was to reach further back into the research cycle and understand, without a research question in mind, why CYP would get involved in research and determine any potential challenges or barriers. By taking this approach, CYP are the leading voice and drive the development of the research design. Therefore, the aim of this project was to identify what information CYP need to know when deciding to take part in research studies using group-based discussion and conversation. This qualitative methodology was chosen because it provides a space for a variety of experiences and points of view to input in a shared activity. Our goal is that this decision-making information will support strategies, confirm that actions are effective, and provide opportunities for increased improvement and justification of actions in future research with CYP living with chronic pain.

## 2 Methods

### 2.1 Your Rheum advisory group

Your Rheum is a national young persons’ advisory group in the United Kingdom (UK) for CYP aged 11–24 years diagnosed with rheumatic conditions. The purpose of the group is to provide CYP with lived experiences of rheumatic conditions meaningful opportunities to input and shape rheumatology research. The Barbara Ansell National Network for Adolescent Rheumatology (BANNAR) founded Your Rheum in 2016 after research led by the BANNAR network investigated not only CYP's research priorities but also how they wanted to be involved in research (20). The Your Rheum story has been documented in detail elsewhere (21); however, the group uses a flexible approach to research involvement regarding both group membership and activity design. The members of Your Rheum/those who signed up for activities can decide which activities they would like to take part in, and through their involvement. BANNAR members who work in Paediatric Rheumatology clinics across the UK are encouraged to invite children and young people to sign up e.g., posters clinical waiting rooms, direct conversations in clinic. The sign-up for Your Rheum and for specific activities is a self-selection process across all of the UK. For each activity offered, 6–10 CYP sign up and attend. CYP do not need to commit to every activity and self-select which activity they are interested in taking part in or because of capacity and costs. As part of this process, CYP gains a better understanding of research and research processes. The process is designed to ensure that the group is inclusive, accessible, and youth-friendly. For example, Your Rheum conducts group and individual research involvement activities that take place in a combination of formats, such as face-to-face and online.

### 2.2 Panel attending video consultation

There were eight young people (female = 7, male = 1, age range 12–24 years) who joined the Your Rheum meeting and took part in the video consultation, sharing their experiences of taking part in research. More than half of the young people had experiences of being a study participant (*n* = 5) and nearly all had previous experience of research involvement via attending a Your Rheum meeting or completing an online activity with the group (*n* = 7). Those CYP who had previously participated in research had mostly taken part in clinical trials and large-scale studies that involved observation/monitoring. Most who took part were recruited for the studies through their parents. Due to how data is collected about the CYP members, we did not have access to ethnicity and demographic information (beyond age and sex) about the panel.

### 2.3 Materials for online session and procedure

**Figure 1:**
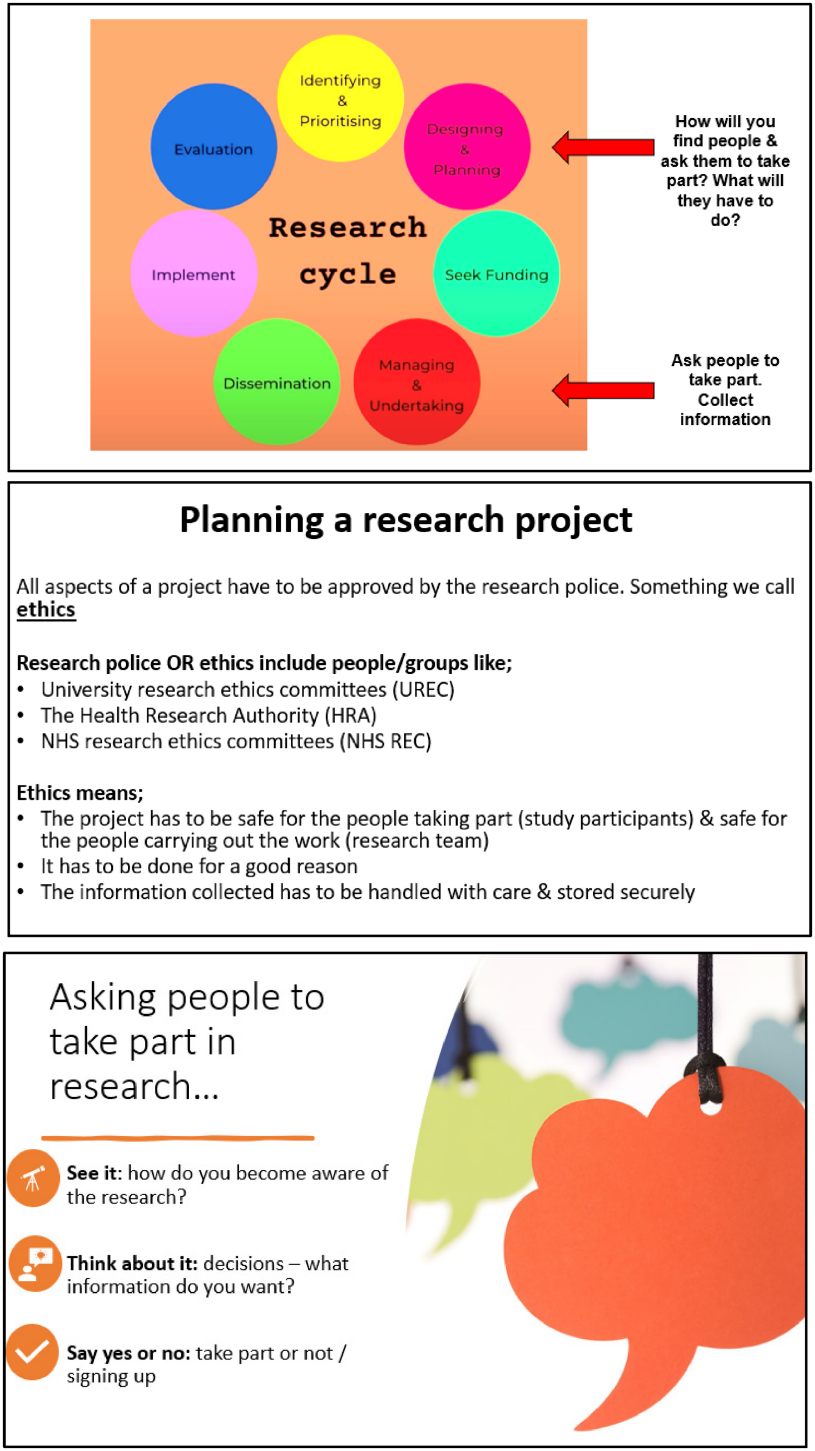
Slides for online meeting.

Advertisements for upcoming Your Rheum online meetings were distributed to BANNAR members, and the team pitched the idea for the current study. The study was then advertised amongst Your Rheum CYP members. CYP receive £20 vouchers and certificates for taking part in virtual Your Rheum group activities. Members of the research team put together slides and activities for the 1-hour virtual meeting with Your Rheum, held via a video conferencing platform. The slides were checked and approved by Your Rheum coordinator, and the consultation was organised through Your Rheum processes (21). This project was designated as a PPI activity. As such, it did not require University ethics approval. Slides (see Figure 1) included an overview of the topic (taking part in research), the research cycle, and the three levels of recruitment; seeing it (exposure), thinking about it (decision process) and confirmation (saying yes or no). Online tools, including Menti-Meter and MIRO, were used to facilitate discussions and share ideas and experiences. Three questions were presented to the CYP. The first two questions focused on thinking about their past experiences when taking part in research, either as a study participant or, if they have never taken part in research, their experiences of joining Your Rheum. For these discussions, the CYP were asked what they would consider before committing to taking part. A word cloud and a decision map were created to reflect the information needs from the perspective of CYP about research projects. The last topic was about the visibility of research in the clinic and during appointments. After the video consultation, the CYP were invited to contact the research team to continue working on the topic to synthesis recommendations from the discussion. Two young people came forward, one who was 24 years old and had undifferentiated inflammatory arthritis and another who was 17 and had rheumatoid arthritis. The young people who approached the team were interested in gaining more knowledge about research processes, such as the reflection and writing aspects of research. One researcher (D.G) wrote reflections from the video consultation and shared them with the research team, including two young people from Your Rheum. With these CYP, we wrote an abstract and a poster (22) with an infographic categorically synthesising the strategies discussed to co-develop five recommendations and steps for recruitment.

## 3 Finding and recommendations

### 3.1 Panel discussions

**Figure 2:**
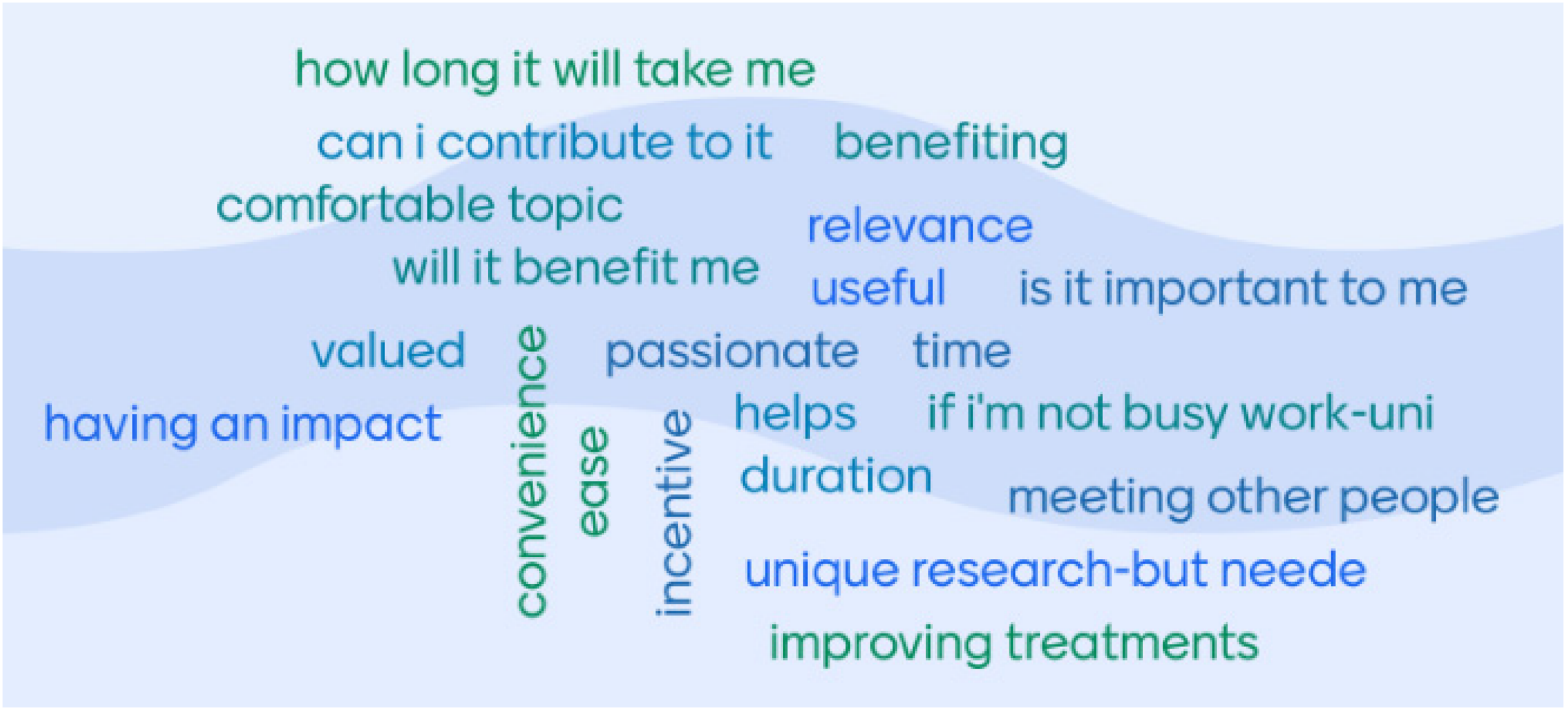
Word cloud depicting CYP considerations about taking part in research.

The word cloud (Figure 2), developed through group discussion, highlighted practical considerations that CYP contemplated when deciding to take part in research (e.g., duration of participation and availability). The second consideration was considering the benefit and impact (e.g., will the research help them/others or will it be a way to connect with others). CYP identified that understanding the benefits of research included finding out more about their condition and being aware of other options for treatment or management, which led to increased disease self-management. CYP felt that taking part in research helped them understand their condition. Discussions about the impact of research focused on exposure to other CYP like themselves, not feeling isolated with their condition and how knowing their condition is being researched, which highlighted to many CYP the importance of their condition and engendered hope for the future.

**Figure 3:**
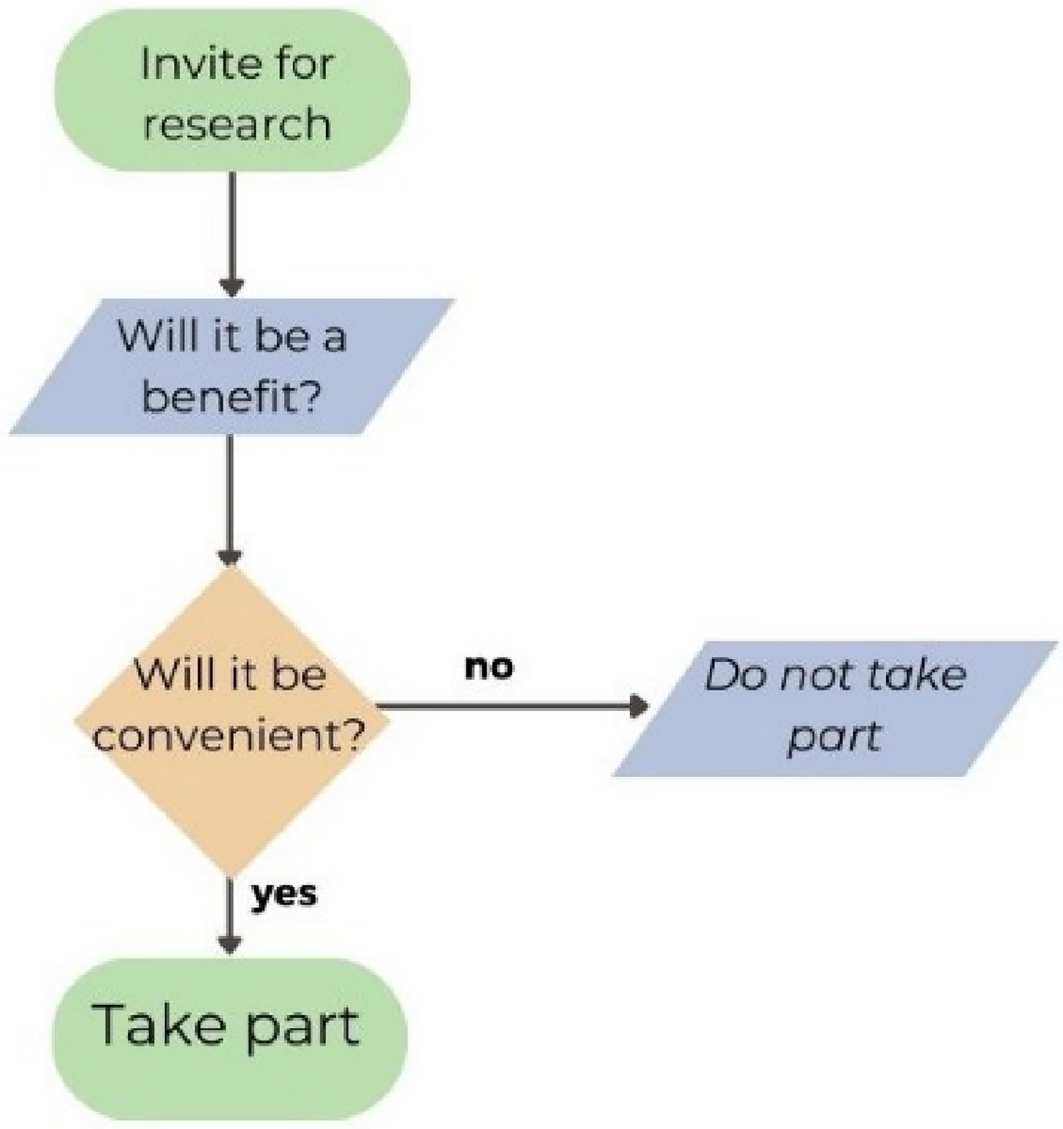
Decision map about taking part in research.

When developing a decision map (Figure 3) together as a group during the video consultation, the first decision discussed was the benefits of research, indicating that this is a high priority for CYP when taking part in research. The next prioritised decision was about the practical considerations regarding convenience/burden or ease in taking part in the research. One young person gave an example of a study that did require a lot of time, but the research team organised transport to and from the hospital, their time was compensated, and they were interested in the topic. These factors contributed to their willingness to participate in the study.

### 3.2 Recruitment recommendations

**Figure 4:**
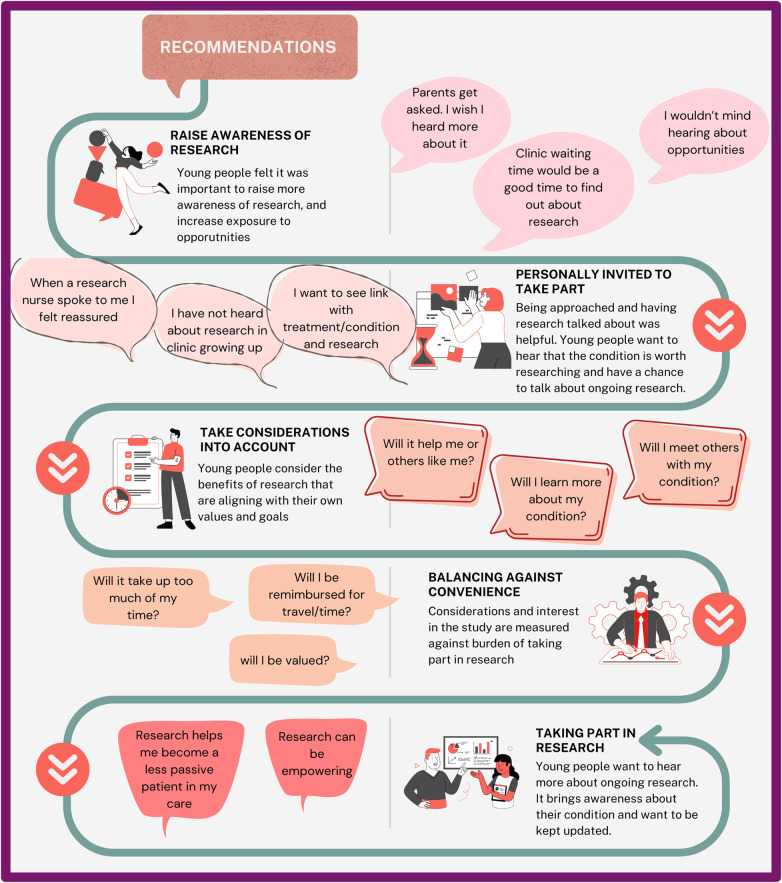
Infographic depicting recommendations for recruitment strategies with CYP.

Findings from video consultation resulted in five coproduced research study recruitment recommendations (Figure 4). These five recommendations were 1. Research awareness (create more visibility of opportunities and exposure to research regardless of eligibility; e.g., being asked and research being discussed with them, using newsletter/emails to circulate research opportunities) 2. Invitations to partake (use of familiar connections or environments, e.g., hospital waiting room, or nurse); 3. Considerations for the benefits of research (create an alignment with CYP values and goals, e.g., will benefit CYP or others like them? Will they meet other young people?); 4. Considerations of personal burden (e.g., is the research convenient, and will the CYP be valued?) and; 5. Impact of taking part in research (e.g., gaining knowledge about the research cycle and empowerment).

### 3.3 Reflections from the young people

The two young people joined the team to co-produce recommendations and steps and provided reflections on the video consultation and the co-production of the infographic.

*“As someone with arthritis, researching different aspects of it (and MSK conditions) is vital to me and participating in it makes me feel like I have an input and possible impact on the future of the condition. Therefore, I wanted to work with the team on this project to further rheumatology research to get more young voices involved in research that is predominately senior-focused*.

*I was surprised at the lack of research discussions at clinic appointments. Being a young person not in paediatrics means that I'm more exposed to research opportunities than most CYP with arthritis. Overall, I found the factors young people include when deciding to participate in research or not, relatable. I agree with most of the factors raised in the discussion and consider the majority of those myself when I'm approached for research opportunities*.

*I enjoyed putting the abstract and poster together. While I've written plenty of academic papers before, nothing outside social sciences so I found the process of contributing to medical research engaging and exciting. Presenting our thoughts and the groups discussions in the most efficient way possible for outsiders to understand was a challenging but ultimately positive.”*

(24 years old undifferentiated inflammatory arthritis.)

*“I wanted to do further work on this project because the project relates to my condition and how it is being researched. I felt that I had never been invited to take part in research, and I wasn*’*t aware of any opportunities that I could take part in. Because of this, I was not surprised when others also mentioned that they were rarely exposed to research opportunities in which they could take part. What did surprise me was that 5 of 7 in the group had taken part in research. I really enjoyed my role in helping to write the abstract, as it was something I had never done before in a topic that interested me.”*

(17, Rheumatoid Arthritis).

## 4 Discussion

Our study examined CYP's decision-making process when taking part in musculoskeletal research. Through this process, CYP identified the paucity of research visibility and awareness. Two young people joined the team to co-develop recommendations in response to the panel discussions. We found that the CYP from Your Rheum advisory panel were enthusiastic about research, discussed the benefits they anticipated from research, and perceived research to be important for them. Despite this interest, discussions with the CYP indicated a lack of exposure and awareness of ongoing research. Because of this deficit, the co-production of the recruitment strategies focused on awareness and exposure of research, which depends on key gatekeepers. In the research context, gatekeepers act as intermediaries with the power to deny or grant access to participation in research studies (23). For research with CYP populations, there are potentially three levels of gatekeepers during research participation (i.e., professional, institutional, and family/caregiver) that must be navigated before the child gains access to information about the study. When there is a reluctance to invite specific CYPs despite eligibility to take part in research, this denies providing CYPs a choice and opportunity, which can be viewed as unethical, and this is then already creating a bottleneck in how we recruit for paediatric pain research. This gatekeeping can emerge from an assumption that the population is vulnerable, but this deprioritizes benefits from taking part in research (24). Gatekeeping was critical to the first co-produced strategy identified in our study as it centred on the visibility of research (i.e., institutional gatekeeping) and increasing awareness about current paediatric rheumatology research, even if this research was not directly relevant to their experience. We recommend ensuring transparency in the sharing of research opportunities and when reporting how we use gatekeeping to reach CYP. Participants suggested using waiting rooms or newsletters for recruitment. However, previous research that has looked at recruitment through posters in GP waiting rooms found that they were not successful in engaging both health professionals and potential adult participants (25), but they are a good location for dissemination of health information and for health promotion (26). However, neither of these previous studies developed the posters with inclusive-PPI input or recruited CYP. The remaining recommendations included mapping onto CYPs' considerations of benefits and reducing burden to reflect their priorities and lastly, about how they want to be part of the process of knowing more about their condition. Therefore, it may be possible, through CYP involvement, to utilize these recommendations and that solutions can be identified to increase exposure through targeted materials for recruitment.

Our second co-produced strategy was related to CYPs being personally invited to research (i.e., professional gatekeeping). In a critical analysis of gatekeeping in CYP's participation in clinical trials, it was argued that gatekeeping becomes unethical and a violation of the CYP's human rights (14) as the right to choose is removed (27). Gatekeeping occurs in all types of research with CYP including paediatric pain research, whilst gatekeeping can help promote recruitment it can exclude groups. We advocate for a universal design approach (materials and process that take into consideration people with a range of abilities and characteristics) that would reduce reliance on gatekeeping (physician decision-making) by closely examining whether study exclusion criteria that do not have scientific rationale are necessary (e.g., language skills of parent/caregiver) (28). Other strategies that are being utilised include “Count Me In,” a novel research recruitment approach used in adult mental health launched in the South of England, where everyone attending centres was given the opportunity to hear about ongoing research unless they opted out of receiving information about ongoing research. Evaluation of this approach has shown an increase in larger cohorts of diverse patients, including more participation from ethnic minoritized groups (29).

Importantly, gatekeeping can also occur before and after research participation. Ethics committees and grant funders may have remits and priorities that exclude some CYP from research participation (e.g., only recruiting from cities in large metropolitan areas; no requirements for diverse and inclusive recruitment). After studies have been completed, dissemination of the findings in peer-reviewed journals may not require inclusive reporting, so the findings can be placed in context, and the limitations are clear. All types of gatekeeping can limit the recruitment and availability of studies for CYP from marginalized groups wanting to take part in paediatric pain research.

Some limitations should be considered when looking at the strategies co-produced from this current project, particularly in relation to the inclusion of marginalised groups. It was not possible to consider a stratified sampling to ensure the panel reflects the diversity of our population. Additionally, Your Rheum advisory panel does not uniformly collect demographic data, such as ethnic minoritized status. Without this data, it is difficult to determine how to improve research participation inclusively. In the next phase of this work, the research team intend to discuss this limitation with Your Rheum to facilitate more inclusive involvement and research awareness. Furthermore, we will ask similar questions to CYP with rheumatic conditions along with another pain condition (e.g., sickle cell disease) to determine if there are differences in recruitment availability for these populations. This future research will focus specifically on CYP from ethnic minoritized groups to understand the precise challenges and barriers along with determining potential solutions to research participation for these underrepresented populations. Although our study provided a unique opportunity to talk about research in general rather than related to a specific project, it would have been good to explore further how CYP envision using newsletters or posters in waiting rooms to make research more visible. This current work has highlighted the importance of research visibility and the need for transparency in our recruitment practices. Our further work in this area will explore this critical area and co-design recruitment material templates to, hopefully, increase recruitment and determine how these tools can be used in paediatric pain research.

The flexible approach in Your Rheum advisory panel allows CYPs to take part in activities in which they feel comfortable, align with their interests, and increase the variety of experiences. Due to the nature of advisory panels in general, there is more awareness of research for the CYP members but also advisory panels like Your Rheum have objectives to learn, teach, and advocate for research (30). That more than half of the young people in this current work have experience of research is reflective of the need for diversity in recruitment for research and for PPI activities. However, despite their involvement in Your Rheum, CYP reflected that they are still not seeing the research opportunities. Given that these CYP represent those with the *most* interest in research, this indicates that much work needs to be done to expand the reach and recruitment of paediatric pain research.

One way to improve inclusivity in research recruitment participation is through community-based participatory research or PPI. Whilst working with CYPs in research is a growing field, we call for researchers to consider the representation of PPI groups, monitor if there is diversity in panels, and explore recruitment strategies utilised for advisory panels, not just for research. Looking at recruitment across all areas of working with CYP, including PPI activities, advisory panels, and research, would help work together on inclusive-recruitment strategies. More recognition is needed that inclusive-recruitment is needed in all these areas, and researchers should also be held accountable for inclusive-recruitment in their PPI partners. The funding body of the UK National Institute of Health Research (NIHR) emphasises and provides the space to report back involvement using the Guidance for Reporting Involvement of Patients and the Public Checklist (GRIPP2) (31); however, there is no guidance on how to report on equity, diversity, and inclusion that would hold researchers accountable in showcasing the efforts made to ensure inclusive-recruitment in PPI activities. However, in a review of NIHR reports of CYP research, only 12% reported against the GRIPP2 (11), so how to report PPI activities and partners effectively should be explored further in paediatric studies. A recent narrative review of techniques used involving CYP showcased how different techniques can address power imbalance and ensure the inclusion of CYP's point of view whilst also assessing how involvement was evaluated. This review highlighted that there is no current standard in the assessment of CYP involvement, and the authors recommend involving young people in the evaluation design (32).

### 4.1 Conclusion

Providing inclusive-recruitment in all aspects of working with CYPs will provide better development of research, will improve recruitment and retention, and provide meaningful evidence. Further work in co-developing inclusive-recruitment with CYPs, specifically CYPs from ethnic minoritized groups, can inform the next steps for paediatric pain research to become more inclusive. The current work is our first step in co-developing recruitment strategies to shift the research landscape for paediatric pain populations.

## Data Availability

The original contributions presented in the study are included in the article/Supplementary Material, further inquiries can be directed to the corresponding author.
